# Social mobilization campaign to tackle immunization hesitancy in Sargodha and Khushab districts of Pakistan

**DOI:** 10.7189/jogh.10.021302

**Published:** 2020-12

**Authors:** Muhammad Nauman Malik, Masood Sarwar Awan, Tariq Saleem

**Affiliations:** 1 Department of Economics, University of Sargodha, Sargodha, Punjab, Pakistan; 2 University of Western Australia, Perth, Western Australia, Australia; 3 District Surveillance Coordinator, Health Department Punjab, Punjab, Pakistan

## Abstract

**Background:**

Immunization hesitancy is a delay in acceptance or refusal of vaccines despite availability of vaccination services. If people are not engaged appropriately via communication and social mobilization, doubts about the trade-offs between the benefits and potential side effects persist. The objective of this study was to explore strategies for improved social mobilization to reduce immunization hesitancy.

**Methods:**

Mix of quantitative and qualitative approaches was applied to collect data from a diverse group of respondents in Sargodha and Khushab districts. Quantitative data were collected from 329 community health workers, including vaccinators, lady health workers and lady health supervisors, and school health and nutrition supervisors. In addition, qualitative data were collected from top management of Expanded Programme on Immunization (EPI) through key informant interviews (KIIs) and focus group discussions (FGDs) were conducted with parents. Analysis has been done using SPSS software and detailed transcriptions.

**Results:**

Advocacy meetings with local influencers, community group sessions, door-to-door visits by community health workers and mosque announcements are considered the most relevant and appropriate interventions for social mobilization. Community Health Workers (CHWs), cognizant of local culture, are being trusted, though optimum performance is achievable with adequate redressal of hesitancy concerns. However, in some cases negative attitudes of people towards immunization hinder trust towards mobilizers or CHWs. Hence, they leverage active participation of local influencers, teachers and health department officials to convince such stubborn parents. Active community involvement through leveraging support from local religious and non-religious influencers in social mobilization activities increases its acceptance. Community engagement is most effective in rural and hard-to-reach areas when community health workers are skilled in interpersonal communication and information education communication.

**Conclusions:**

Communication committees as oversight mechanism should be established or reactivated to regularly monitor and support mobilization activities through managing affairs like speedy liaison with local administration and local influencers, mobilizers’ service related concerns, community-specific hurdles, and deficiencies of awareness-material provision that eventually improves mobilization performance. Resistant community’s needs can be redressed through rigorous conduct of men’s and women’s education sessions by CHWs while giving more time and space to mobilizers to take on board local religious and non-religious influencers to convince conservative/illiterate parents. Higher management should fix policy implementation slippages like training needs assessment of mobilizers and Civil Society Organizations’ involvement framework.

In Pakistan, the rate of fully immunized children remained at 82% during the period 2014–2015 against the national target of 90% [1], and in its most populist Punjab province the rate remained at 81% [2]. Immunization hesitancy exists among people in different parts of Pakistan. Blatant refusals are diminishing and now are being replaced with ‘immunization hesitancy’ in which parents are weighing the arguments for and against vaccination, which is observed in South Asia [3]. This hesitancy may not be addressed by the demand generation or mobilization activities of the Expanded Programme on Immunization (EPI) [4]. Hence, it is important to identify system failures in social mobilization campaign as an intervention against immunization hesitancy which is based upon various social and economic reasons [5].

Under-coverage of immunization in Pakistan is caused by both demand and supply factors such as poverty, competing family priorities, perceived benefits from the health services, acceptability of immunization services, problems with the outreach services and availability of services [4].

Immunization hesitancy refers to the delay in acceptance or refusal of vaccination despite availability of vaccination services. It is complex and context specific, varying across time, place and vaccines, and influenced by factors such as complacency, convenience and confidence [6-9]. Interventions that tackle demand-side challenges include knowledge generation and awareness-raising activities, communication campaigns and provision of incentives to seek care [10]. Lack of awareness and misconception eliminates the demand for immunization of children, especially in deprived and marginalized groups [11]. Furthermore, it is observed that on the ground, EPI staff face many difficulties in flood prone areas, and problems with security issues, incorrect understanding of vaccinations in areas or within groups with low socio-economic and education levels [12]. Social mobilization campaigns that are sufficiently planned, funded and integrated with service delivery can help in immunization coverage [13]. The success of vaccination programmes depends upon people having sufficient knowledge to make an informed decision to receive appropriate vaccines [14,15]. Immunization decision-making is a complex process susceptible to many factors. If people are not engaged appropriately through appropriate design and implementation of social mobilization activities for generating demand, doubts about the trade-offs between the benefits and harms of vaccination and fears about side effects persist [16-20]. Knowledge and awareness through successful intervention often increase coverage of child immunization [21,22]. It is required to assess social mobilization campaign of EPI for its good practices and deficiencies.

The overarching objective of this study was to explore strategies for improving social mobilization campaign activities to decrease immunization hesitancy. This study particularly explored issues around **appropriateness/relevance, acceptability and fidelity** of social mobilization campaign activities.

## Methods

### Theoretical framework

**Figure 1:**
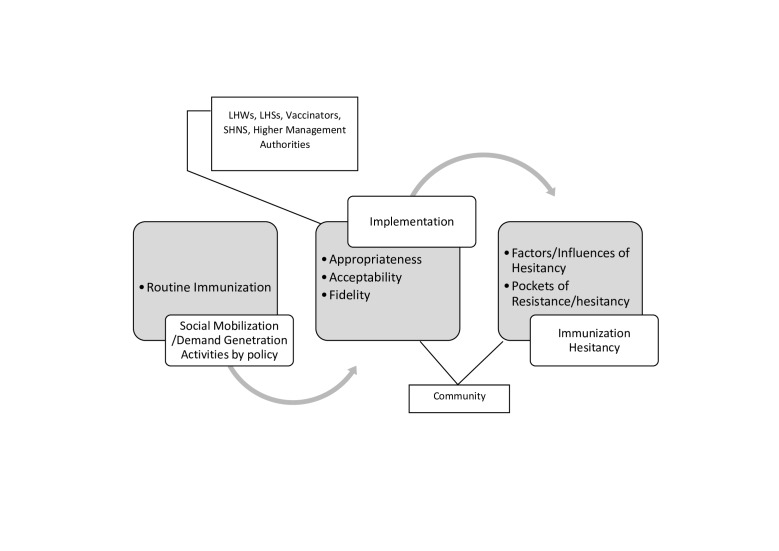
Social mobilization activities, implementation and their impact on immunization hesitancy (authors’ own illustration).

For this research, we applied a theoretical framework illustrating that the demand generation activities of EPI staff at grass root level works as an intervention to decrease immunization hesitancy if intervention is appropriate to context, acceptable and as per given policy (fidelity) [23-25]. **Figure 1** shows that at implementation stage, higher management, CHWs and community are involved that serves as a conduit for legislation to pursue immunization hesitancy. ‘Appropriateness’ is judged by the intensity of appropriateness of mobilization activities to the local context of immunization hesitancy. ‘Acceptability’ is judged by the trust that people conferred on the local mobilizers, community involvement in activities and the acceptance of new changes in the programme by CHWs. ‘Fidelity’ tells whether policy document had been adhered or not. We aimed to ask CHWs (LHW, LHS, Vaccinators and SHNS) and parents (community) about appropriateness and acceptability, and higher management and CHWs about fidelity (**Figure 1**).

### Study design

Both quantitative and qualitative approaches were used to collect data from a diverse group of respondents. Semi-structured questionnaire was used to collect data from different stakeholders involved in a social mobilization campaign at the union council level, such as school health and nutrition supervisors (SHNSs), lady health supervisors (LHSs), lady health workers (LHWs) and vaccinators. Union Council (UC) is a lowest administrative hierarchical level where EPI and Basic Health Unit (BHU) staff are supposed to conduct their mobilization activities. Questionnaire for CHWs covered the topics of the degree of appropriateness for mobilization activities in the local context, acceptability and trust that people conferred on the local mobilizers, community involvement in activities, and the acceptability of new changes in programme by CHWs. In addition, questions were asked about adherence, frequency of activities, quality of performance, effectiveness of collaborators in relation to their envisioned role, and suggestions for improvement.

In addition, Focus-Group Discussions (FGDs) were conducted with community members to find out their general perception regarding the overall appropriateness and acceptability of mobilization activities. This helped in the interpretation of the survey results and was needed to triangulate the FGD findings to gain a more meaningful interpretation of survey results. Semi-structured Key-Informant Interviews (KIIs) were conducted with the higher management of EPI in the provincial capital city of Lahore, which provided information on what has been achieved and what has not in relation to national and provincial communication policy implementation. These in-depth interviews primarily focused on questions related to policy implementation. Questionnaire tools of survey, FGDs and interviews were built after going through past literature of immunization, social mobilization, implementation research and policy documents.

### Study sites

The study sites were Sargodha and Khushab districts of Punjab Province, where survey and FGDs were conducted. The full immunization coverage rates in Sargodha and Khushab districts are 68% and 66%, respectively [1]. Sargodha and Khushab districts are composed of 7 and 4 *tehsils* (sub-districts), and 161 and 51 union councils, respectively [24]. Each of the union councils is provided with at least one BHU.

### Sampling and sample size

For quantitative survey four types of respondents (SHNS, LHS, LHW and vaccinator) from each BHU were interviewed. Fifty BHUs were selected, each in a different union council from both Sargodha and Khushab districts, by using the probability proportional to size (PPS) methodology as the sampling technique for the survey (**Table 1**).

**Table 1:** BHUs and type of respondents in Sargodha and Khushab districts

Details of BHUs				Details of type of respondents				
**Sr. No.**	***Tehsil* (sub-district) name**	**Total BHUs**	**Selected BHUs**	**SHNSs**	**LHSs**	**LHWs**	**vaccinators**	**Total**
Sargodha District								
1	Sargodha	45	20	20	19	20	19	78
2	Sillanwalli	16	6	6	6	6	6	24
3	Shahpur	12	4	4	4	4	4	16
4	Sahiwal	12	5	5	5	5	5	20
5	Kot Momin	16	7	7	7	7	7	28
6	Bhalwal	14	6	6	6	6	6	24
7	Bhera	9	2	2	2	2	2	8
Sub-total Sargodha District		124	50	50	49	50	49	198
Khushab District								
1	Khushab	16	23	0	13	23	23	59
2	Quaidabad	07	08	0	4	8	8	20
3	Noorpur Thal	12	11	0	7	11	11	29
4	Naushera	8	8	0	7	8	8	23
Sub-total Khushab District		43	50	0	31	50	50	131
Total Sargodha and Khushab districts		167	100	50	80	100	99	329

BHU – basic health unit, SHNSs – school health and nutrition supervisors, LHSs – lady health supervisors, LHWS – lady health workers

The aim was to have for each selected BHU, one respondent from each type of four respondents, which held true for Sargodha District. However, in Khushab District, the planned respondents fell short due to different reasons including vacant positions of SHNS and more than one BHU under the same LHS. Convenience sampling was used for FGDs and KIIs. One FGD comprised of 10 members was conducted with community members/parents having children less than 5 years of age in each district, along with two interviews with the higher management of EPI Punjab in Lahore.

### Data collection and analysis

Survey of health workers was undertaken in august and september of 2017. Pretesting of the questionnaire was done in two BHUs (one in each district) and with all four types of respondents in each BHU. The closed ended questions were provided with options such as Very poor, Poor, Average, Good and Very good, or Don’t use; Very rarely, Rarely, Occasionally, and Frequently; or Not effective, Least effective, Moderately effective and Most effective. Responses of open-ended questions were coded later for analysis. Post graduate students of University of Sargodha, Pakistan under the team leadership of faculty members conducted face-to-face interviews of health workers in field survey. Later, data editing was done with subsequent entry of it in SPSS computer software package. Later, the analysis of quantitative data was completed in terms of percentage measure of the responses. One of us (MNM) conducted both FGDs and KIIs but (MSA) remained in FGDs only to initiate and as listener in discussions. Both have no conflict of interest or bias given their professional and personal affinities. We conducted two FGDs of 10 participants/parents who have children of less than 5 years age in each district’s randomly chosen union council. We solicited help from union council’s councillor (political representative) to work as field mobilizer and asked him to randomly get acceptance of parents for participation in FGD. On the specified day, we randomly chose 10 parents from the list of parents who gave willingness to councillor for participation. FGDs and KIIs were recorded and later transcriptions were cross-checked by an expert in public health and implementation research, who was neither in FGDs and KIIs nor had any conflict of interest with EPI organization and survey areas. FGDs brought out opinion of community about implementation (**Figure 1**). The two KIIs with the officials of higher management of EPI were semi-structured interviews which were devised to ask about the implementation of prevalent national communication strategy for immunization [25] (**Figure 1**). Same procedure was adopted for the interpretation of KIIs as was for FGDs. The result’s section is fairly distributed among the three implementation research assessment parameters; appropriateness, acceptability and fidelity [23]. Appropriateness would tell us about the relevance of different social mobilization activities with suggestions; Acceptability would inform acceptability level of CHWs by the community, level of community involvement in social mobilization activities with suggestions to increase the potential of CHWs to adapt to new changes in activities. Fidelity means the degree to which an intervention was implemented as it was designed in an original protocol, plan or policy. National Communication Strategy for Routine Immunization Pakistan would be our benchmark to check implementation at community level.

### Ethical approval

Ethical approval was obtained from the Institutional Review Board (IRB) of Health Services Academy (HSA), Islamabad to conduct this study. Prior to each quantitative interview, FGD and KII, verbal consent was obtained from the respondents.

## RESULTS

The results section is divided in three themes covering study’s objectives named as appropriateness, acceptability and fidelity. Each theme is further sub-divided in evidences.

### Appropriateness or relevance of the social mobilization activities

**Figure 2:**
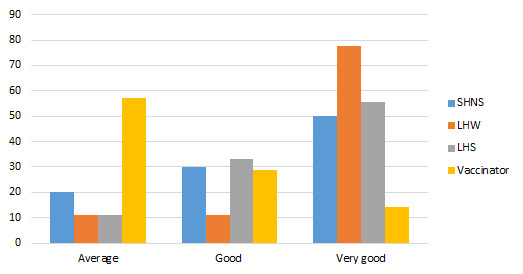
Appropriateness of advocacy meetings to tackle immunization hesitancy (%).

Among the four types of respondents in this study, the majority of SHNSs (50%), LHSs (55.6%) and LHWs (77.8%) consider advocacy meetings as a very good strategy, while the majority of vaccinators (57.1%) rated it average against immunization hesitancy (**Figure 2**).

**Figure 3:**
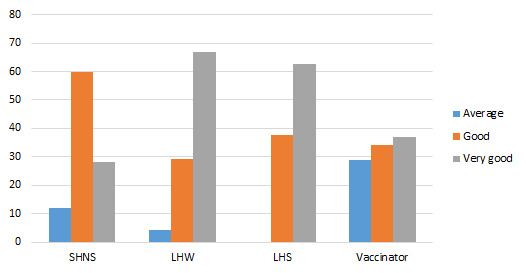
Appropriateness community group meetings/sessions to tackle immunization hesitancy (%).

All categories of respondents are in favour of community meetings (**Figure 3**). 66.7% LHWs, 62.5% LHSs and 36.8% vaccinators reported it as very good and 60% SHNS and 34.2% mentioned it as good.

**Figure 4:**
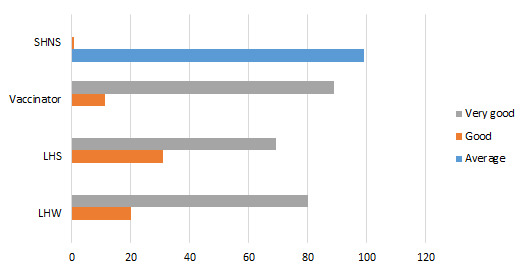
Appropriateness of door-to-door visits for tackling immunization hesitancy (%).

**Figure 4** shows door-to-door visit is considered as one of the best strategies by 88.9% vaccinators, 69.2% LHSs and 80.0% LHWs as very good and 99% SHNS mentioned it average in sensitizing community members to immunization hesitancy.

100% respondents endorsed that health session activity as a very good strategy to address immunization hesitancy.

**Figure 5:**
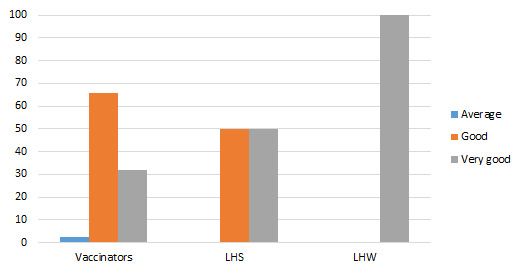
Appropriateness of announcement in a mosque (%).

100% LHWs and 50% LHSs rated it as a very good strategy. 65.9% vaccinators endorsed it as good one (**Figure 5**). Overall, in FGDs, there was a common voice of endorsement of social mobilization activities as appropriate and relevant.

**Figure 6:**
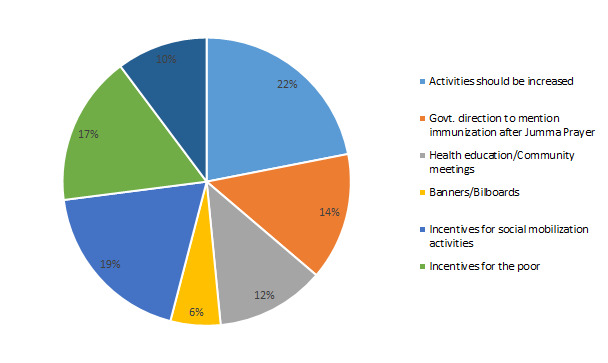
Suggestions for improving relevance of activities in the local context, percentage of respondents (%).

To improve relevance, 22% of the total respondents in quantitative survey suggested to increase the frequency of social mobilization activities (**Figure 6**).

About 14% of the respondents mentioned that government should explicitly issue orders to the imam *masjids* (mosques) to explain the importance of immunization after Friday *(Jumma*) prayers so that people are reminded to abide by the schedule of the vaccination cards and vaccinators’ visits to the local community. About 12% of the respondents favoured introducing community health sessions in which the local health facility team give health education on regular basis. In FGDs, there was the suggestion to use a notable person from the local area for such health education sessions where men, exclusively, are invited. As mentioned by one of the FGD participants:

“…the government can use the presence of a notable person from the local area for health education lectures where community people should be invited”.

About 19% of the respondents suggested increasing funds for conducting social and community gatherings and meetings either in the budget of BHU or specific to EPI. Steps for demand generation in certain impoverished groups, such as the brick kiln community, riverine tenants and nomadic people, need to be specifically targeted to persuade them to receive immunizations. 17% of the respondents said to give these groups of people both monetary and non-monetary incentives to stick to their schedules of vaccinations.

### Acceptability

43% and 39.7% of the respondents mentioned that the level of community trust towards health workers is good and very good, respectively. As one FDG participant responded,

“We trust them (Community health workers)!”

There are various reasons for this. Some 61.3% of the respondents mention that because SHNS, LHS and LHW belong to the local area, community trust towards them is strong. One of the FGD participants explained about a social mobilization staff member:

“Yes, we know him (Community health workers) and he even meets us while moving in or out of our local area as he is native and from our local area”.

Benefits of originating from the local community also include the same local language, customs and traditions. On the question of whether local women understand the way of knowledge/information deliverance, one of the FGD participants answered,

“Yeah! Our women understand their language and talking style, and they understand what is being communicated to them because we have same language.”

Other trust-winning factors mentioned include experience (19%), good reputation (11%) and good behaviour (9%). About 16% of the respondents felt that trust is very poor where negative attitudes toward vaccinations encourages immunization hesitancy. More than 80% of the responses fall in the category of customary norms, which covers disinterested behaviour towards government workers due to general dissatisfaction with the government. It also covers the issues where vaccinators of particular *baradari* (Extended network of relatives belonging to same caste and usually living in the same area) are not acceptable or where vaccinators are from a particular *baradari* that has any local area conflict or strife with the targeted area population.

**Figure 7:**
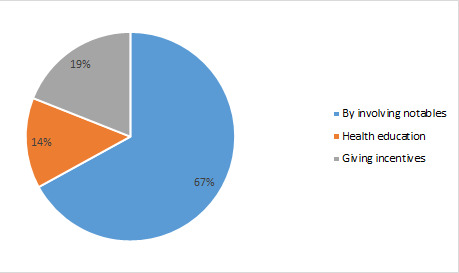
Remedies to overcome low levels of trust and acceptability (%).

As a means to increase immunization coverage, it was suggested by 67% of the health workers to involve more notable people from the local area (**Figure 7**). At the same time, 14% said conducting periodic health sessions communicating health-related information.

One of the FDG participants admitted,

“In villages, we have ignorance. Therefore, open publicity should be done as other departments do, like the agriculture department’s education extension service. There should be health education awareness.”

Majority of the CHW respondents endorsed the fact that community involvement is satisfactory and impressive (**Table 2**). As one of the FDG participants mentioned,

**Table 2:** Community involvement

Level of community involvement in the social mobilization campaign as per the view of respondents (%)		Reasons (%)		
Very Poor	16.5	Low involvement	Stubborn/illiterate individual	41.6
			Busy in personal work	41.6
			Either want repeated requests or incentives	16.9
			Total (approximately) of respondents answering ‘Very poor’	100
Average	28.3	High involvement	Awareness	46.7
Good	23.7		Native identity	26.7
Very good	31.6		Experience	17.5
			Good reputation	4.2
			Good behaviour	5.0
			Total (approximately) for respondents answering ‘Average’, ‘Good’ or ‘Very good’	100

“We all help and support them whatever they (EPI staff) want to do. There is no hurdle from the community.”

People who are more aware about immunization, usually individuals who are more educated, share the same native identity and have good social reputation and behaviour, have high involvement in social mobilization activities.

About 42% respondents mentioned that people, including educated people, appear to be busy in personal work, and are not particularly interested in EPI staff activities or concerned with the general awareness and benefit of the community.

**Figure 8:**
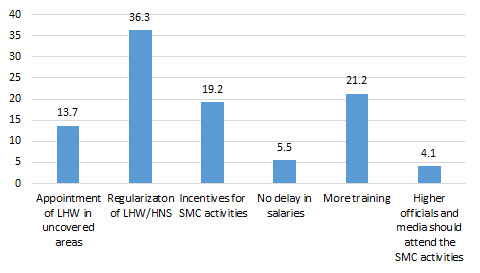
Suggestions for Improvement in the acceptability and adaptability of new changes in social mobilization activities (%).

Several strategies were suggested to increase the capacity and adaptability of health workers to accept new changes in social mobilization activities (**Figure 8**), such as no delay in the disbursement of salaries (5.5%), regularization of jobs (36%), and appointment of LHWs in areas where they are not posted (13.7%).

Another suggestion from respondents (21%) for improvement was that more trainings should be done for CHWs.

### Fidelity

**Figure 9:**
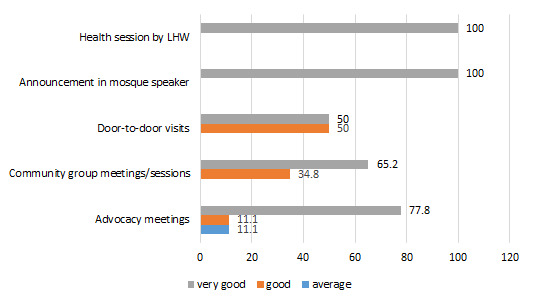
Performance of social mobilization activities (lady health worker's opinion) (%).

Among the different mobilization activities as prescribed by National Communication Strategy for Routine Immunization Pakistan, advocacy meetings, community meetings, announcements using mosque speakers, health sessions and door-to-door visits are currently being adhered by our respondent CHWs (**Figure 9**).

All respondent LHWs and LHSs mentioned that health sessions organized by LHWs and mosque announcements about immunization were considered as very good strategies to tackle immunization hesitancy (**Figure 9**). Regarding door-to-door visits and organization of community group sessions, more than 50% of the LHWs, and about 39% of LHSs found these very good strategies, whereas 57% of the vaccinators found these very good strategies. About 77% of the LHWs, 73% of the LHSs and 57% of the vaccinators found advocacy meeting as very good social mobilization strategy to tackle immunization hesitancy.

**Figure 10:**
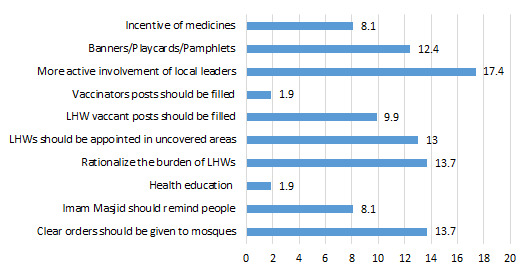
Suggestions for improvement in the performance of social mobilization (%).

As far as general suggestions for improvement of social mobilization activities (**Figure 10**), 17% of the CHWs suggested more active participation of local social leaders to overcome immunization hesitancy. About 2% and 10% of the respondents suggested increasing the number of vaccinators and LHWs by filling vacant posts, respectively. About 13% of respondents suggested appointing LHWs in those areas that are still out of reach and currently uncovered. FGDs revealed,

“You should convey the message of appointment of LHWs in areas where they are not working.”

About 14% of respondents suggested rationalizing the burden of LHWs, as it is very hard for them to conduct social mobilization activities in the field. Almost 14% suggested that mosques should be given clear orders to help health facility staff whereas 8.1% said that imam masjid should be made liable for reminding community members about the importance of immunization. About 12% suggested the number of pamphlets, banners and posters should be increased so that they can be displayed in the other places in the community in addition to in the BHUs.

On the question of CSO engagement, one official explicitly and comprehensively stated,

“Very few! We may have with one or two [CSOs] only! In my view, they don’t work so much. The experience of other provinces in this domain is not good and so we didn’t go after them. They usually pull out from the field after six months to one year and the whole structure [of interaction and support with them] comes down.”

It is proposed to develop specific material to engage religious leaders, politicians, health care workers etc [24]. There is already the practice of writing material with a focus on polio; however, there is no such practice of developing specific material for groups on ‘regular basis’ that can help address broader immunization issues at the social and community level. As one official said,

“*Special written material is prepared only when there is some high level of resistance or refusal from the area.*”

Similarly, it was recommended that material be developed emphasizing the importance of retaining vaccination cards, which are the only recorded means of adherence to vaccination schedules at home and serve to inform health and socio-economic surveys.

KII exposed about updating of an animated series as,

“Yeah! we have this MEENA film *[animated film]* on routine immunization BUT it is old material *[episodes]* and not new. We show the old material to people”.

## DISCUSSION

This study tested social mobilization implementation against low knowledge, misdirected awareness, and socio-economic destitution whom are instilling immunization hesitancy as pointed by various studies [11,12,26-29]. This study found that advocacy meetings, community meetings, mosque announcements about immunization sessions or mentioning of immunization significance in periodic religious sermons, door-to-door visits and health sessions were appropriate and relevant to the local context of immunization hesitancy. These similar activities gave promising outcomes in India’s context too [30]. Moreover, various reviews on mobilization activities, analysing varying country-settings, proposed the same what we practically observe in our findings [31-35].

Some of the community-level suggestions and study results justify the **appropriateness and relevance** of social mobilization tools including advocacy meetings and community meetings to address immunization hesitancy of people belonging to different ideas, backgrounds, ethnic identities, geographic area groups, occupation groups etc. as envisaged by the policy [25]. Such meetings for *dairajaat* (geographic isolated group of people from main dense village settlements and/or uncovered areas where LHWs are not working) and resistant communities were conducted by EPI/BHU staff in support of one another. Local influencers visit households known to resist or hesitate, along with EPI staff. This finding is in tune with the success of ‘*Bullawa Tollies’* tactic in India [36]. In some instances, support from local administrative/revenue department was arranged.

LHWs and LHSs organized health committees/support groups to streamline door-to-door visits and health sessions [29]. Door-to-door visits are very much relevant in the local context because often women are not being permitted by their husbands and/or in-laws to leave house during day time to attend women’s meetings/health sessions arranged for awareness. Women in isolated communities like *dairajaat,* brick kiln worker communities or nomadic groups are difficult to gather (especially in the absence of any monetary or non-monetary incentives) for group awareness sessions because they are busy in their own work and live in far-flung areas [37]. Additionally, there are various so-called religious beliefs prevalent in local areas that do not allow pregnant women to come out of house or to speak with others outside home, even in the early months after birth. Such reasons make these women the most vulnerable cases where awareness and information should be provided at home to ensure proper information on health care and immunization coverage. This finding of door-to-door visits’ relevance is in tune with the revelations about Uganda and Lao PDR where husband’s non-cooperative behaviour prevents correction of suspicions among their women [38,39].

Health sessions come under Integrated Reproductive Maternal New born Child Health Programme and they are helpful in reducing hesitancy. Here, our study found little contrasting results than that of Uganda [38]. Health sessions are appropriate. The environment of the health session is more intimate than ‘community meetings’ and more dialogue-oriented than ‘door-to-door visits’ as they allow for cross-discussion among women, LHSs and LHWs.

Mosque Announcements about immunization sessions and the significance of immunization are found to be appropriate and relevant, as many people are motivated by religious instruction. This tool also gained success for immunization coverage in India [36]. However, in some areas, this means cannot be used as *imam masjids* (local religious leaders) say that it is forbidden by the government to use the loudspeaker for anything other than *azan* (wake up call for Muslims to offer prayer in mosques). It is reported that often the announcement is not made in all the mosques related to various sects and sub-sects in the Islamic religion. Hence, the people belonging to a sect may get annoyed if the announcement is not made in the mosque of their sect. Here, community health workers try to conduct meetings with all local sectarian religious leaders using their personal connections in community to convince them about the significance of immunization.

In terms of **acceptability,** this study found that primarily native identity of community-level mobilization staff helped them to win trust of people along with good behaviour and experience in line with the findings for Venezuela where trust over EPI servicemen improve immunization coverage dismantling hesitancy [40]. Moreover, low community involvement in mobilization activities is often from illiterate people such as nomadic or migrated *pathan*/Afghan families, who are the least interested in attending mobilization activities. Part of the reason may be their extreme deprivation and poverty. To increase more community links, this study found that more time allocation for social mobilization in routine working hours and resources are required, as also prescribed in WHO study [41]. This study found that LHWs’ involvement is still hailed as the most acceptable new change, since the inception of EPI in 1978 [42].

In the context of **fidelity**, this study found that activities like advocacy meetings, community meetings, mosque announcements, health sessions and door-to-door visits are being adhered on regular basis. Such adherence was not being done in similar way for SMS/voice messages, marketing on transit vehicles, local radio, public service messages in local cinemas and on TV, animated series update on CDs, and banners and billboards. Such material’s exposure is crucial for immunization coverage rise in consonance with what had been found for India [43]. Among other envisaged social mobilization policy initiatives, this study found that communication committees had not been established whereas such committees’ oversight is essential to ensure the quality and impact of communication activities as seen for Afghanistan, Nigeria and India [44]. Civil Society Organizations (CSOs) had not been taken on-board through any formal structure. One reason might be of inconsistent nature due to their non-sustainable financial resources but in stark contrast to what in recent times Malawi did that it encouraged its non-governmental organizations which performed well in social and community mobilizations and produced bright results [45,46]. Training needs assessments have not been conducted yet. Monitoring of social and community mobilization activities is just on ad hoc basis. In uncovered areas, vaccinators could more easily become successful if LHWs were working in those areas. Sadly, still uncovered areas exist. Immunization significance had not been incorporated in the curriculum of primary schools as suggested in policy. LHWs needed written material with proper pictorial description to be used in health sessions and community meetings. Efforts for mobilization of resources from business community were not implemented as envisioned in policy [25]. Hence, last but not the least, these highest level policy implementation slippages compromised the total efficacy of communication strategy and many policy parameters remained far off from the implementation stage.

This study is the first one in local contexts to assess social mobilization campaigns to combat immunization hesitancy based on the criteria of implementation research outcomes, ie, appropriateness, acceptability and fidelity. Our findings corroborate the argument that ‘package’ of intervention including both mobilizers and complementary-materials of mobilisation can win against immunization hesitancy [47-49].

## Conclusion

Our study recommends some tactical systemic initiatives to improve the social mobilization implementation for routine immunization of EPI.

Communication committees as support mechanism should work to analyse community-specific mobilization needs both related with mobilizers and complementary mobilization material. This mechanism can support and give more flexibility to mobilizers in making liaison with local area religious and non-religious influencers to overcome hard-core resistance pockets. At the same time, rigorous and successful conduct of education sessions by CHWs for men and women would also be possible with that liaison. Hard-core hesitant areas can be focused by these communication committees. They should micro-manage existing mobilization resources including CHWs to target those resistant communities. They can be of great help in stirring up local administration and health department attention to solve specific concerns eg, less deployment of LHWs in uncovered areas and lack of real-time local administration’s instructions for political and non-political officials to help mobilizers. Use of media, public service messages, brochures, pamphlets and descriptive-cum-pictorial materials should be ensured to enable LHWs to disseminate vaccinate-related information and encourage retention of vaccination card. Such complementary materials increase mobilisation efficacy of CHWs. Education sessions should be organized with conservative and illiterate segments of the population. More time and financial resources should be reserved to implement social mobilization activities in resistant areas, and it can be possible with active vigilance of communication committees. Training needs assessment of CHWs involved in social mobilization should be conducted to arrange relevant trainings of CHWs to enhance their capacity beyond native identity’s comparative advantage. CSOs should also be taken on board as supportive agent for social mobilization. Policy initiative slippages regarding training needs assessment, CSO engagement framework and liaison with businesses for funds mobilization from the higher management should be avoided in future.
